# The Diversity and Molecular Evolution of B-Cell Receptors during Infection

**DOI:** 10.1093/molbev/msw015

**Published:** 2016-01-22

**Authors:** Kenneth B. Hoehn, Anna Fowler, Gerton Lunter, Oliver G. Pybus

**Affiliations:** ^1^Department of Zoology, University of Oxford, Oxford, United Kingdom; ^2^Wellcome Trust Centre for Human Genetics, University of Oxford, Oxford, United Kingdom

**Keywords:** molecular evolution, B-cell receptor, diversity, immunoglobulin, infection.

## Abstract

B-cell receptors (BCRs) are membrane-bound immunoglobulins that recognize and bind foreign proteins (antigens). BCRs are formed through random somatic changes of germline DNA, creating a vast repertoire of unique sequences that enable individuals to recognize a diverse range of antigens. After encountering antigen for the first time, BCRs undergo a process of affinity maturation, whereby cycles of rapid somatic mutation and selection lead to improved antigen binding. This constitutes an accelerated evolutionary process that takes place over days or weeks. Next-generation sequencing of the gene regions that determine BCR binding has begun to reveal the diversity and dynamics of BCR repertoires in unprecedented detail. Although this new type of sequence data has the potential to revolutionize our understanding of infection dynamics, quantitative analysis is complicated by the unique biology and high diversity of BCR sequences. Models and concepts from molecular evolution and phylogenetics that have been applied successfully to rapidly evolving pathogen populations are increasingly being adopted to study BCR diversity and divergence within individuals. However, BCR dynamics may violate key assumptions of many standard evolutionary methods, as they do not descend from a single ancestor, and experience biased mutation. Here, we review the application of evolutionary models to BCR repertoires and discuss the issues we believe need be addressed for this interdisciplinary field to flourish.

## Introduction

The adaptive immune system ensures the survival of humans and other vertebrates in the face of rapidly evolving and genetically diverse infectious diseases. B lymphocytes are an essential component of this system and express receptors on their cell surface (B-cell receptors; BCRs) capable of specifically binding foreign antigens. BCRs are membrane-bound immunoglobulins composed of two large heavy chain molecules and two smaller light chain molecules, encoded in humans by the genes *IGH* and *IGL* (or *IGK*), respectively. The diversity of BCRs expressed by an individual’s B cells is vast, and comprises both naive receptors that are randomly generated from the germline during development, as well as receptors that are retained after successfully binding antigen during previous infections. Populations of BCRs can rapidly improve antigen binding during infection through an evolutionary process of mutation and selection known as affinity maturation (Liu et al. 1991). Because BCR sequences are diverse and diverge rapidly, concepts from molecular evolution should be beneficial in understanding the dynamics of the adaptive immune system within individuals. T-cell receptors (TCRs) are a second class of immune receptors that can bind foreign antigen. Although diverse TCRs are also generated randomly from germline gene sequences, and their comparison with BCRs can be illuminating, TCRs do not undergo affinity maturation nor exhibit rapid evolution during infection. Therefore in this review, we focus solely on BCR biology.

Apart from molecular assays that characterize sequence length polymorphisms (e.g., TCR immunoscope assays; see Bercovici et al. 2000) there was, until recently, a paucity of data on within-individual BCR sequence diversity for researchers to explore. That situation has now changed with the application of next-generation sequencing to BCRs (Boyd et al. 2009; Six et al. 2013). With this technique, researchers can directly observe the somatic genetic changes that generate the diversity of the BCR repertoire, providing an unprecedented picture of the adaptive immune system as an evolving population of cells. Analyses of these data from an evolutionary perspective have led to insights into the aging of the B-cell repertoire (Wang, Liu, Xu, et al. 2014) and into the process of affinity maturation (Elhanati et al. 2015; Yaari et al. 2015), and have many applications across a broad range of diseases (see table 1).

**Table 1:** **.** Applications of BCR Repertoire Sequencing.

Application	Diseases/Infections	Significance	Challenges
Identification of broadly neutralizing antibodies (BNAbs)	Infection with rapidly evolving pathogens such as HIV, Hepatitis C virus, influenza viruses	Potential for use as vaccine targets (Haynes et al. 2012) Provide model system for understanding affinity maturation, coevolution, and immune development (e.g., Wu et al. 2015)	Lineages are often large and diverse, often with high levels of hypermutation, long CDR3s, and poly reactivity (Haynes et al. 2012)
Study of vaccine responses	Any disease for which vaccines are used or being developed, e.g., Influenza, typhoid, or Ebola	A model system for immune response with a known stimulus (Galson et al. 2014) Identifying correlates of immune protection following vaccination	Distinguishing vaccine-specific changes to the BCR repertoire from healthy repertoire diversity Variable immune responses among individuals to the same stimulus (Ademokun et al. 2011; Jiang et al. 2013)
Tracking B-cell migration and development within the body	Autoimmune diseases such as multiple sclerosis; cancers	Identifies migration of B cells between tissue compartments (von Büdingen et al. 2012) Can identify the sites at which B cells mature (Stern et al. 2014)	Accurate B-cell lineage assignment Modeling potentially complex migration patterns Differential sampling between tissues
Disease diagnosis	Autoimmune diseases such as multiple sclerosis and rheumatoid arthritis; cancers, in particular B-cell lymphoma	A direct and potentially cheap diagnosis tool Improved understanding of disease Provide clinical markers of disease progression (Robinson et al. 2013)	Complex and multiple disease epitopes induce complex responses Low level presence of B cells associated with disease

Despite these advances, there are important challenges in applying models and methods from molecular evolution to BCR sequences, which stem both from the complex biology of B cells and the nature of available data. Unlike most natural populations, naïve BCR sequences do not descend from a single common ancestor through a process of point mutation, but are instead generated from a diverse set of germline gene segments through a process of somatic recombination (see below). In addition, the mutation process during affinity maturation is strongly dependent on the sequence context of flanking nucleotides (Yaari et al. 2013) and selection on the resulting amino acid sequence is complex and site-specific, driven by the need to avoid dangerous self-reactivity while concurrently enhancing pathogen binding. Finally, BCRs are a complex of heavy and light chain immunoglobulin molecules, and information from both is necessary for a complete understanding of BCR evolution and function. Here, we review the current literature on these topics and explore how molecular evolution and phylogenetics may contribute to future BCR research.

## B-Cell Development

**Fig. 1:**
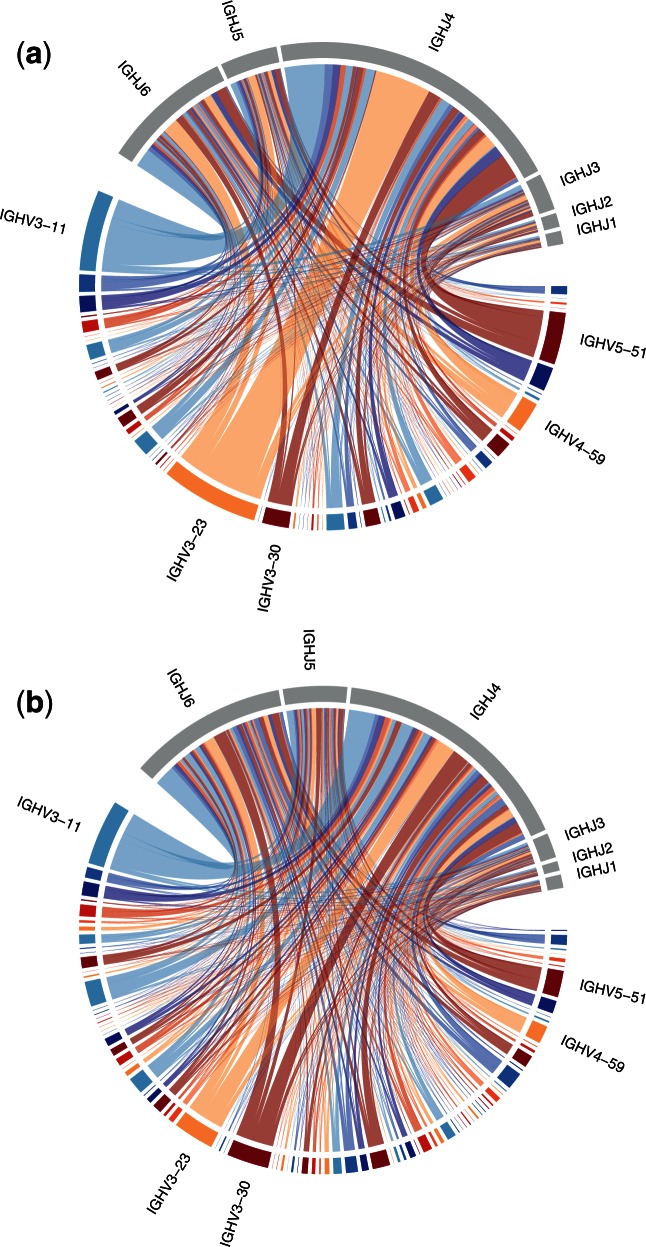
Chord diagrams showing the pairing of V and J segments within (*a*) productive and (*b*) nonproductive IgM sequences from a single healthy individual. Chord widths represent the proportion of sequences with a given V (colored) and J (gray) segment pairing. The five most common V segments in productive rearrangements (and all J segments) are labelled. Note that *IGHV3-23/IGHJ4* was significantly more common in productive versus nonproductive rearrangements, which may indicate functional bias of that pairing. The figure was generated from data in Elhanati et al. (2015), which was aligned to the IMGT reference (Lefranc et al. 2009) using IgBLAST (Ye et al. 2013). Productive rearrangements were subsampled to the same read depth as nonproductive rearrangements (∼2 × 10^5^ reads); the values displayed in (*a*) are means of 100 subsampling repetitions.

The initial diversity of the BCR repertoire is the result of a somatic recombination process called V(D)J recombination. This process brings together one each of the variable (V), diversity (D), and joining (J) segments of the *IGH* locus on chromosome 14 to form an exon in the heavy chain immunoglobulin gene, and one each of the V and J segments of the *IGL* (or *IGK*) locus to form the light chain. Not all gene segments are utilized. Of the 123–129 *IGHV* gene segments, 44 contain open reading frames (ORFs); further, 25 of the 27 D segments and 6 of the 9 J segments have been shown to be used for somatic recombination in the heavy chain (Lefranc M-P and Lefranc G 2001; Li 2004). During this process, additional sequence diversity is generated by random deletion or insertion of nucleotides at segment junctions. This process combines highly variable sequence regions that determine antigen binding (the complementarity determining regions; CDRs) with more conserved framework regions (FWRs) that provide structural support. Thus each naïve B cell has its own BCR sequence, and the number of possible BCR sequences is huge, with models predicting at least 10^18^ (Elhanati et al. 2015), far greater than the number of B cells in the body. The process may generate nonproductive (e.g., out-of-frame) coding sequences; when this happens, the B cell may recombine its second copy of the *IGH* gene. If this too fails to produce a viable recombinant sequence then the cell undergoes apoptosis, which further modulates the background genetic diversity of receptors (fig. 1). The surviving, naïve B cells then undergo an initial round of selection for lack of self-reactivity, before they are released from the bone marrow into peripheral blood (Murphy et al. 2008).

Once a naïve B cell is activated by binding a foreign antigen, it undergoes cell division (clonal expansion) and initiates processes that somatically alter the BCR sequence and diversify the clonal population. In parallel, a mechanism called class switching alters the constant region of the heavy chain, changing the type and function of the BCR and its interaction with other molecules; although this does not affect its antigen-binding properties, it does leave a molecular mark that can be used to separate naïve BCRs from those that have undergone affinity maturation. Affinity maturation modifies antigen binding through a process of random and rapid sequence change, termed somatic hypermutation (SHM), and by selection. SHM involves greatly increased mutation rates of approximately 10^−^^3^ changes per nucleotide per cell division, corresponding to approximately one mutation per cell division in the relevant locus (Teng and Papavasiliou 2007; Victora and Nussenzweig 2012). Mechanistically, these mutations are induced by the enzyme activation-induced cytodine deanimase (AID), which deaminates cytosine to uracil during transcription (Muramatsu et al. 2000; Teng and Papavasiliou 2007; Peled et al. 2008). Importantly, for evolutionary analysis, SHM is a random and strongly nonuniform process, and clearly distinct from the processes of germline mutation and evolution. In particular, SHM is context-dependent such that the probability of mutation at a site is strongly influenced by neighboring nucleotides (Shapiro et al. 2003; Yaari et al. 2013; Elhanati 2015). The resulting mutations are further shaped by a round of selection, in which B cells compete for survival and replication signals by competitively binding to antigens (Peled et al. 2008). The combination of these processes shapes both the type and rate of observed mutations across the *IGH* and *IGL* loci.

## Sequencing the BCR Repertoire

The extraordinary variability of BCR sequences poses challenges for targeted sequencing. We provide here only a brief summary of current sequencing approaches, in particular as they relate to the analysis of BCR diversity. Rearranged VDJ segments are flanked by introns, so targeting germline DNA requires a cocktail of polymerase chain reaction (PCR) primers (Larimore et al. 2012). A challenge for this approach is to control for PCR bias, which could skew the frequency of sequenced variants and obscure the signal of clonal expansion. An alternative approach that can significantly reduce the problem of PCR bias is to target expressed mRNA, in which case the constant regions flanking the VDJ segments in mature mRNA can be used for PCR priming (Galson et al. 2015). In addition, different classes of B cells can be distinguished by targeting different constant regions. The challenges for mRNA sequencing are to 1) disentangle variation in sequence frequency that is due to differential expression, which can be extensive, from that due to clonal expansion; and 2) ensure that sequencing error and subsequent bioinformatic processing do not introduce systematic biases into subsequent evolutionary analyses. For a more detailed discussion of BCR repertoire sequencing, see the reviews by Benichou et al. (2012) and Robins (2013).

Sequencing of the somatically altered heavy chain has the potential to reveal the clonal structure and dynamics of the B-cell population through time, and this review focuses on the analysis of bulk sequence data from this region. However, although the majority of variation in BCR sequences is concentrated in the heavy chain, and in particular the CDRs (Xu and Davis 2000; Georgiou et al. 2014), the light chain also contains mutations that may affect antigen binding. If one’s goal is to characterize entire antibodies, or to understand the binding properties of a given heavy chain sequence, then knowledge of paired heavy and light chain sequences is required. Computational approaches have previously sought to infer how heavy and light chain sequences are paired from independently sequenced sets of sequences by using relative frequencies (Reddy et al. 2010), or the shapes of phylogenetic trees (Zhu et al. 2013) of heavy and light chain sequences. Recently, single-cell technologies have enabled natively paired heavy and light chains to be sequenced by attaching unique barcodes to cDNA from individual cells (Busse et al. 2014; Lu et al. 2014; Tan, Blum, et al. 2014; Tan, Kongpachith, et al. 2014). Alternatively, oligo-dT beads that link heavy and light chains from a single cell have been used (DeKosky et al. 2013, 2015).

## Measuring BCR Diversity

Once BCR sequences are generated, statistical and computational approaches are necessary to explore and summarize their diversity, in order to reveal associations with immune responses or disease status, or to identify BCR sequences of specific interest. The exceptional diversity of the BCR repertoire, and its dynamic nature, makes comparative study within and among individuals challenging.

**Fig. 2:**
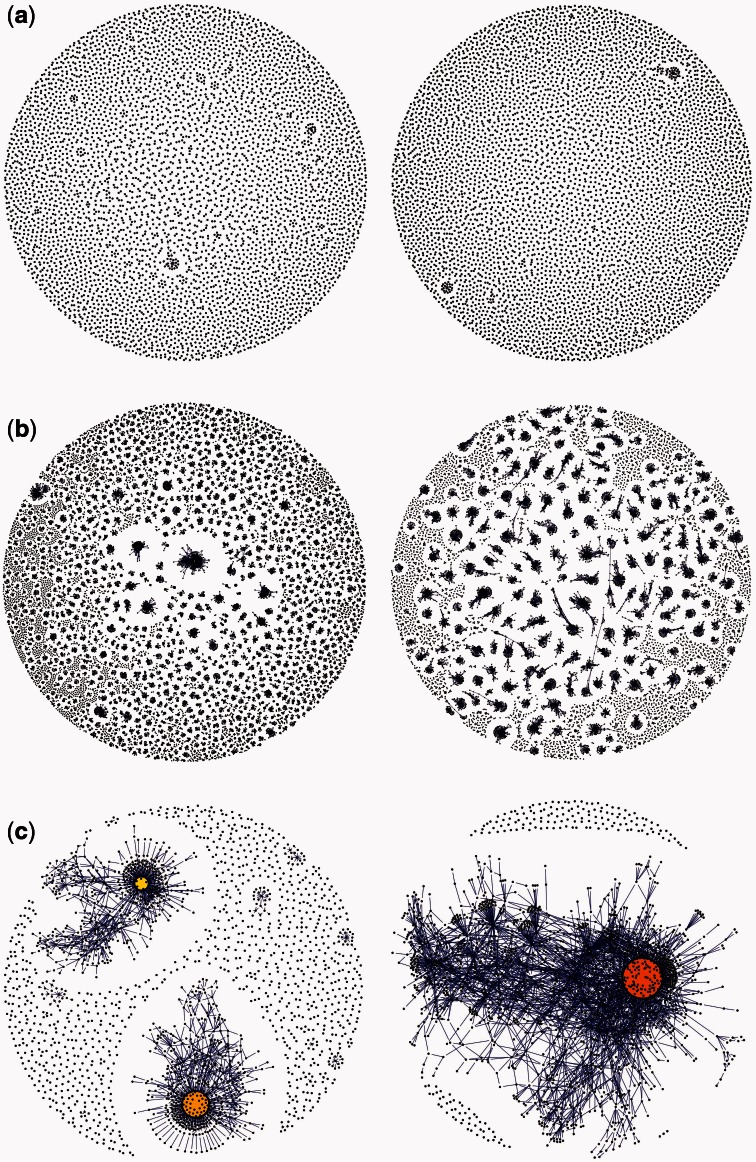
Network diagrams that visualize the diversity and clonal structure of BCR sequences. BCR sequences were obtained from (*a*) two healthy people, (*b*) two individuals infected with HIV-1, sampled during early infection, and (*c*) two patients with chronic lymphocytic leukemia, a B-cell cancer. Each point/circle represents a unique BCR sequence, the size of which is proportional to how common that sequence is. Edges are drawn between pairs of sequences that differ by exactly one nucleotide change. Note that samples do differ by read depth (approximately 3.4 × 10^4^ and 3.6 × 10^4^ for part [*a*], 9.2 × 10^4^ and 3.6 × 10^5^ for part [*b*], 5.1 × 10^4^ and 2.6 × 10^4^ for part [*c*]). Parts (*a*) and (*c*) are reproduced with permission from Bashford-Rogers et al. (2013) and part (*b*) from Hoehn et al. (2015).

Several different measures have been proposed, and can be distinguished into those that characterize raw sequence variability versus those that depend on the frequency of BCR lineages, clones or clusters (i.e., groups of identical or similar sequences; see next section). In the context of viral infection, both the number of somatic mutations (Chen et al. 2012; Wang, Liu, Xu, et al. 2014; Galson et al. 2015) and V, J gene usage (Zhou et al. 2013) have proved useful. CDR3 sequence length also has been used to distinguish repertoires after pneumococcal vaccination (Ademokun et al. 2011; Chen et al. 2012; Galson et al. 2015). Diversity statistics such as the Gini index or mean clone size are also used to investigate BCR diversity (Bashford-Rogers et al. 2013; Galson et al. 2015; Hoehn et al. 2015). Figure 2 provides a graphical representation of BCR diversity under different conditions.

Other approaches seek to characterize BCR diversity using statistical models. Mora et al. (2010) introduced a maximum entropy model that characterizes the repertoire as a statistical distribution, whereas Elhanati et al. (2015) used probabilistic inference to quantify the process of VDJ recombination and SHM. Greiff et al. (2015) proposed employing entropy measures developed in ecology research, which unify a range of diversity measures into a single profile.

A common assumption of these approaches is that clonal expansions observed in infected individuals correspond to B-cell responses against the pathogen under study. This may not always be true, especially in instances of coinfection with multiple pathogens. Further, some infections may manipulate host immune responses through so-called superantigens (e.g., staphylococcal protein A), which trigger large, nonspecific clonal expansions that disrupt antigen-specific affinity maturation (Thammavongsa et al. 2015). Such phenomena do not in general prevent clonal expansions from being useful indicators of immune dynamics, but require them to be carefully interpreted in the context of the particular host–pathogen system.

Comparison of BCR populations among individuals is of interest because repertoires may become similar if individuals are exposed to the same pathogen, giving rise to a shared, “public” repertoire. Differences in BCR repertoires between individuals are likely generated by many factors including age (Wang, Liu, Xu, et al. 2014), germline genetics (Wang, Liu, Cavanagh, et al. 2014), and infection history (Sasaki et al. 2008; Wang, Liu, Xu, et al. 2014). Infectious diseases typically present many epitopes, and even when different B cells target the same epitope, it is possible for the different BCR sequences to bind equally effectively. Despite these complexities, BCR convergence following identical stimuli has been observed, and has enabled the identification of antibodies reactive against influenza vaccines (Jackson et al. 2014; Martins and Tsang 2014; Trück et al. 2015) and dengue virus vaccines (Parameswaran et al. 2013). In addition, antibodies against HIV that exhibit the same broadly neutralizing phenotype, and which share some common sequence elements, have evolved independently in different patients (Scheid et al. 2011; Zhou et al. 2013). It is currently an open question whether convergent molecular evolution of BCR sequences is a common or an exceptional phenomenon (Yaari and Kleinstein 2015). Some tests of convergence have been developed in other contexts, such as Zhang and Kumar’s (1997) convergent evolution hypothesis test, which directly compares substitution models of convergent versus independent evolution along preselected lineages. This and other methods designed to detect convergence (e.g., Parker et al. 2013) may improve our understanding.

## Clonal Lineage Assignment and Clustering

Molecular phylogenetics is an undeniably powerful tool for analysis of sequence diversity. However, its application to BCR repertoires is impeded by the V(D)J recombination process, the existence of which means that not all BCR sequence differences are due to point mutation through descent from a common ancestor. Consequently, sequences must be grouped by lineage, each representing sequences that descend from a single ancestral B cell, before they can be analyzed phylogenetically (Hershberg and Luning Prak 2015).

A key step in this process is the alignment of BCR sequences to reference data sets of V, D, and J gene segments, in order to determine their germline origin. Several such alignment methods are available: IMGT/High-V-Quest (Alamyar et al. 2012) is popular and provides a well-curated reference data set; IgBLAST (Ye et al. 2013) can be run with a user-specified reference data set, and IgSCUEAL uses phylogenetic relationships between germline genes to increase the accuracy of assignment (Frost et al. 2015). Other tools include iHMM-Align (Gaeta et al. 2007) that implements a hidden Markov model, and VBASE2 (Retter et al. 2005) that uses a reference data set provided by Ensembl. Other techniques, using methods adopted from phylogenetic ancestral state reconstruction, assign V(D)J segments while also quantifying uncertainty in assignment (Kepler 2013). However, germline V, D, and J segments vary considerably among individuals and new alleles are still being discovered, so the reference data set may be inaccurate (Gadala-Maria et al. 2015). Segment similarity, junctional diversity, SHM, and sequencing errors all further increase the difficulty of unambiguously assigning BCR sequences to specific germline segments. This is particularly true for D segments, due to their short length (11–37 nucleotides; Lefranc M-P and Lefranc G 2001; Giudicelli et al. 2005) and the frequent occurrence of deletions during V(D)J recombination, which may remove part or all of a D segment (Elhanati et al. 2015).

**Fig. 3:**
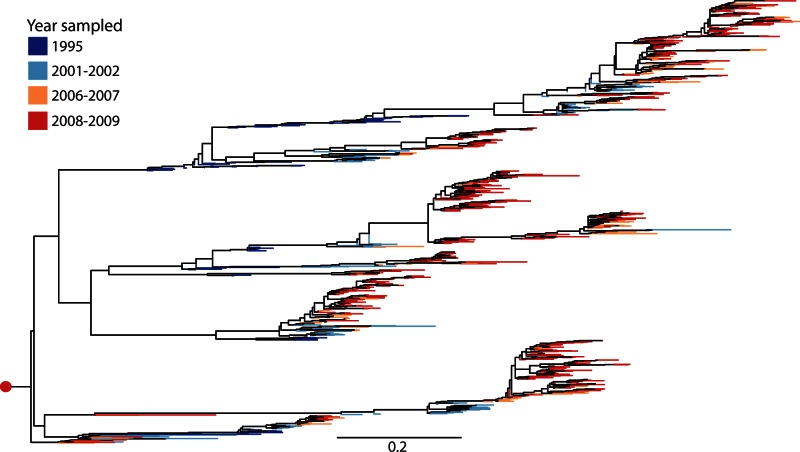
Maximum-likelihood phylogenetic tree of the VRC01 BNAb lineage sampled at ten time points over 15 years of diversification within a single individual infected with HIV-1. Each tip represents a BCR heavy chain sequence; terminal branches are colored by time point of sampling (see key). The red circle at the root represents the germline sequence (IGHV1-2*02 and IGHJ1*01, D region left unassigned). Note the general, but not complete, trend of increasing genetic divergence from the root with sampling time. Late-sampled sequences near the root indicate very high rate heterogeneity among lineages; these sequences might represent inactive memory B cells. BNAb sequences were obtained through cell sorting (Wu et al. 2010) followed by high-throughput sequencing data to identify related BCRs (Zhou et al. 2013). See Wu et al. (2015) for full experimental details. Sequences for this tree were obtained from GenBank (Wu et al. 2015) and aligned using MUSCLE (Edgar 2004). A maximum-likelihood phylogeny was estimated using the GTRGAMMA substitution model in RAxML (Stamatakis 2014), and rerooted to position the germline sequence at the root with a divergence of zero. Scale bar represents genetic distance (expected changes per nucleotide site).

Several studies sidestep the problem of germline assignment, and instead use clustering approaches to group similar BCR sequences by using either the entire V(D)J region sequence (fig. 2; Bashford-Rogers et al. 2013; Hoehn et al. 2015) or the CDR3 region (Jiang et al. 2013; Sok et al. 2013; Laserson et al. 2014). A threshold number of differences (edit-distance) is often used to determine whether sequences belong to the same or different clusters. One difficulty with this approach is the choice of threshold. Some studies address this by exploring multiple thresholds; edit-distances of three to five differences have been chosen by looking at how cluster numbers and sizes change as the threshold is increased (Yaari et al. 2013; Laserson et al. 2014). However, a more principled approach is clearly needed to test how closely these clustering techniques reconstruct the true clonal structure of the B-cell population. At present they appear well suited for the detailed analysis of recently diverged clones (Laserson et al. 2014), and for quantifying the diversity of the BCR repertoire in general (Bashford-Rogers et al. 2013; Hoehn et al. 2015; Trück et al. 2015). However, it is unlikely that clustering approaches based on edit-distances will be effective in accurately identifying large, diverse lineages. For example, broadly neutralizing HIV lineages often show high levels of genetic diversity (fig. 3; Wu et al. 2015), and the intermediate (i.e., ancestral) sequences necessary for accurate clustering may not be available in many cases, because affinity maturation occurs in the germinal centers rather than in peripheral blood (Parham 2009). Studies that have successfully isolated large and diverse B-cell lineages from HIV-infected patients have generally done so using by combining sequence analysis with detailed experimental work (Zhou et al. 2013).

## Untangling Mutation and Selection

**Fig. 4:**
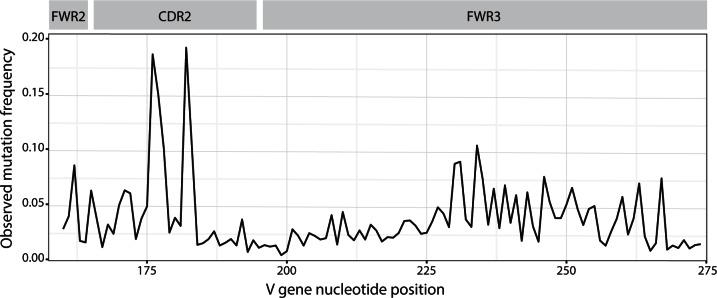
Observed mutation (sequence difference from germline) frequency among productive heavy chain immunoglobulin sequences across the V-gene sequence (horizontal axis, IMGT unique numbering). The distribution of mutations across the region is strongly nonuniform, with mutations more likely to occur at certain positions. The CDR2 region (middle shaded box) has a high rate of observed mutations and is thought to be more important in antigen binding than the surrounding framework regions (FWR2 and FWR3). This figure was generated from the same data set as figure 1.

The enzyme-driven nature of SHM poses a challenge for studying the molecular evolution of BCRs. Standard nucleotide substitution models typically assume that sites (either nucleotides or codons) evolve independently (Felsenstein 1981). However, SHM is strongly context dependent, to the extent that observed mutation rates vary more than 10-fold across sites (see fig. 4) (Elhanati et al. 2015). Consequently, traditional methods for identifying positive and negative selection that rely on uniform-rate independent-site models can generate false positives when applied to BCR sequences, for example, within nonproductive (out-of-frame) sequences that are not subject to selection (Dunn-Walters and Spencer 1998).

Models of SHM based on empirical data have been developed and include di-, tri-, penta-, and hepta-nucleotide models (Smith et al. 1996; Shapiro et al. 2002, 2003; Yaari et al. 2013; Elhanati et al. 2015), and have been used to investigate selection on the naïve B-cell repertoire (Elhanati et al. 2015). Selection has been explored using the “focused” binomial test, which determines whether the observed number of replacement mutations is significantly different from that expected under a null model of biased mutation but no selection (Hershberg et al. 2008). This framework has subsequently been extended using Bayesian inference (BASELINe; Yaari et al. 2012). In common with other components of the acquired immune system (e.g., class I and II *MHC* glycoproteins; Yang and Swanson 2002; Furlong and Yang 2008), analyses indicate that BCR sequences are a mosaic of regions under a mixture of positive and purifying selection (i.e., CDRs) and structural regions whose evolution is highly constrained by purifying selection (i.e., FWRs) (Yaari et al. 2012, 2015; McCoy et al. 2015).

It should also be possible to detect the action of antigen-driven selection from the shape of BCR lineage phylogenies, which represent the common ancestry of a sample of sequences from a lineage of clonally related B cells (Dunn-Walters et al. 2002). Computer simulation of lineage trees generated by affinity maturation under a variety of scenarios found seven measures of tree shape that correlated strongly with immunological parameters (Shahaf et al. 2008). However, recent analyses using these measures concluded that they are affected by experimental factors that are difficult to control, such as the number of sequences sampled from a lineage and the number of cell divisions since initial VDJ rearrangement (Uduman et al. 2014). Utilizing lineage information, such as excluding terminal branch mutations, has been shown to increase the sensitivity of methods based on the expected number of replacement mutations (Uduman et al. 2014). It is interesting to note that very similar approaches were developed independently in viral phylogenetics, specifically in studies of HIV-1 and influenza populations under strong positive selection (e.g., Bush et al. 1999; Lemey et al. 2007).

Recently, two further approaches to analyzing B-cell selection have been developed. Kepler et al. (2014) used a statistical model of selection and an empirical model of sequence mutability to study their interplay along the BCR sequences of an antibody lineage. Alternatively, one can adjust and control for the motif-targeted nature of SHM by studying and comparing productive and nonproductive BCR rearrangements within a given data set (see “B-Cell Development”; Larimore et al. 2012; Elhanati et al. 2015; McCoy et al. 2015). McCoy et al. (2015) combined this information with a statistical model of trait evolution (Lemey et al. 2012) in order to derive a per-residue map of natural selection along the BCR.

Although antigen-driven positive selection is of great interest, of equal importance to the evolution of antigen-specific BCR sequences is the influence of purifying selection, which results from the removal of self-reactive and nonproductive receptors and which partly precedes the affinity maturation stage (see B-Cell Development). This initial selection can be studied by comparing the mutation profiles of nonproductive and productive BCR sequences; the latter often have shorter CDR3 sequences postselection, and exhibit complex and position-dependent selection for and against particular amino acids (Elhanati et al. 2015).

## BCR Phylogenetics

The process of affinity maturation generates rapid sequence evolution, so it is unsurprising that phylogenetic approaches are now routinely used to visualize how B-cell lineages undergo diversification and divergence in response to an antigen. Phylogenies have been used to address important problems, such as reconstructing ancestral BCR sequences within a lineage (Kepler 2013; Sok et al. 2013), detecting and measuring selection on B-cell populations (Uduman et al. 2014), and studying how broadly neutralizing antibodies sometimes evolve in response to HIV infection (Wu et al. 2015). Further integration of phylogenetic concepts, including those from fields such as viral phylodynamics (Grenfell et al. 2004; Volz et al. 2013), may improve our understanding of affinity maturation dynamics during infection. For example, the rate of SHM evolution in a lineage over time (and its variability among lineages) could, in theory, be revealed by using molecular clock models to analyze BCR sequences sampled at different times. Further, asymmetric tree shapes might help to identify the action of strong positive selection on serially sampled antibody lineages (fig. 3), analogous to phylogenetic footprint left by recurrent selection on some influenza virus lineages (Grenfell et al. 2004). However, as noted in the previous section, BCR lineage tree shapes may be subject to biases that are not yet fully understood (Uduman et al. 2014), so for the time being they should be interpreted with caution.

Although many phylogenetic analyses focus exclusively on BCR heavy chain sequences, the light chain may also be included, for example, by concatenating the two gene sequences together (Wu et al. 2015). As both chains are inherited together during B-cell replication, they should share the same phylogenetic topology. By adding more sites to the alignment, concatenation may improve the accuracy of phylogeny estimation (Huelsenbeck et al. 1996; Gadagkar et al. 2005). However, if the mode or tempo of molecular evolution differs between heavy and light chains, then it may be advisable to divide the concatenated sequences into separate partitions, each with its own molecular clock and nucleotide substitution model (e.g., Nylander et al. 2004)

However, current phylogenetic models may not represent adequately the particular processes of growth and mutation that generate BCR lineages and therefore they should be applied with caution. For example, Wu et al (2015) recently used a relaxed molecular clock model to analyze the evolution of a broadly neutralizing antibody lineage (VRC01) sampled over 15 years of HIV-1 infection. The estimated date of the common ancestor of the lineage was implausibly old, which led the authors to conclude that the molecular clock model used was unrealistic. Specifically, they concluded that the mean rate of BCR evolution of VRC01 and other lineages (Liao et al. 2013; Doria-Rose et al. 2014) slowed over the course of lineage development. This work poses interesting avenues for future research, as it should be possible to test the slowdown hypothesis directly using a time-dependent molecular clock model. Alternatively, the apparent slowdown could be caused by the AID motif-driven nature of BCR mutation, in which case fundamental assumptions of the nucleotide substitution model (e.g., independence among site and time-reversibility) may be inappropriate. It is likely that current evolutionary models will need to be substantially modified or carefully selected before we can be confident in evolutionary inferences from BCR sequence data.

## Conclusion

BCR sequence data contain a wealth of novel immunological information and have the potential to improve our observation and understanding of the mechanisms of autoimmune disease and acquired immunity (table 1). However, the dynamic processes that determine the response of B-cell populations to diverse antigens differ from other forms of biological evolution in key ways, some of which are currently poorly understood. We conclude by outlining four important challenges facing the molecular evolutionary analysis of BCR sequences.

1. **Distinguishing between biological signal and experimental error or bias.** Many aspects of experimental protocol may have an effect on observed sequence diversity, including read depth and length, PCR conditions and primers, and cell sorting. Close collaboration between experimentalists and analysts is needed to ensure that experimental choices are appropriate for subsequent evolutionary analyses.
2. **Identifying clonally** **related cells/sequences.** For evolutionary methods to be maximally informative, it is necessary to distinguish within-individual BCR sequence differences caused by SHM from those derived from V(D)J recombination. Advances here might include improvements in sequencing or experimental protocols; development of methods to probabilistically cluster into clonal lineages; and the creation of a “gold standard” test data set allowing evaluation of methods for determining clonal lineages.
3. **Detecting convergent evolution among B cells responding to the same stimulus.** The prevalence and importance of this process, and its utility for understanding the underlying biology of BCRs, is currently under debate. Improved understanding of the frequency distribution of naïve BCR sequences should help to estimate the fraction of the public, shared repertoire that occurs by random chance. In addition, it may be possible to adapt methods from molecular evolution and phylogenetics to make progress in this area.
4. **Models to describe the process of BCR affinity maturation.** Although descriptive summary statistics have proven useful for the visualization and qualitative analysis of BCR repertoires, further understanding will be gained by developing stochastic process models that embody the known mechanisms of SHM and B-cell proliferation, and by the application of such models to empirical data. Finally, it is important to understand the potential biases arising from applying standard phylogenetic and molecular evolutionary models to BCR sequences. These could be investigated by analyzing artificial BCR data sets simulated under complex and biologically realistic models of sequence evolution.

## References

[msw015-B1] Ademokun A Wu Y-C Martin V Mitra R Sack U Baxendale H Kipling D Dunn-Walters DK. 2011 Vaccination-induced changes in human B-cell repertoire and pneumococcal IgM and IgA antibody at different ages. Aging Cell 10:922–930. 21726404 10.1111/j.1474-9726.2011.00732.x PMC3264704

[msw015-B2] Alamyar E Duroux P Lefranc M-P Giudicelli V. 2012 IMGT® tools for the nucleotide analysis of immunoglobulin (IG) and T cell receptor (TR) V-(D)-J repertoires, polymorphisms, and IG mutations: IMGT/V-QUEST and IMGT/HighV-QUEST for NGS. Methods Mol Biol. 882:569–604. 22665256 10.1007/978-1-61779-842-9_32

[msw015-B3] Bashford-Rogers RJM Palser AL Huntly BJ Rance R Vassiliou GS Follows GA Kellam P. 2013 Network properties derived from deep sequencing of human B-cell receptor repertoires delineate B-cell populations. Genome Res. 23:1874–1884. 23742949 10.1101/gr.154815.113 PMC3814887

[msw015-B4] Benichou J Ben-Hamo R Louzoun Y Efroni S. 2012 Rep-Seq: uncovering the immunological repertoire through next-generation sequencing. Immunology 135:183–191. 22043864 10.1111/j.1365-2567.2011.03527.x PMC3311040

[msw015-B5] Bercovici N Duffour M-T Agrawal S Salcedo M Abastado J-P. 2000 New methods for assessing T-cell responses. Clin Diagn Lab Immunol. 7:859–864. 11063487 10.1128/cdli.7.6.859-864.2000 PMC95974

[msw015-B6] Boyd SD Marshall EL Merker JD Maniar JM Zhang LN Sahaf B Jones CD Simen BB Hanczaruk B Nguyen KD, 2009 Measurement and clinical monitoring of human lymphocyte clonality by massively parallel V-D-J pyrosequencing. Sci Transl Med. 1(12):12ra23. 10.1126/scitranslmed.3000540 PMC2819115 20161664

[msw015-B7] Bush RM Bender CA Subbaro K Cox NJ Fitch WM. 1999 Predicting the evolution of human influenza A. Science 286:1921–1925. 10583948 10.1126/science.286.5446.1921

[msw015-B8] Busse CE Czogiel I Braun P Arndt PF Wardemann H. 2014 Single-cell based high-throughput sequencing of full-length immunoglobulin heavy and light chain genes. Eur J Immunol. 44:597–603. 24114719 10.1002/eji.201343917

[msw015-B9] Chen W Prabakaran P Zhu Z Feng Y Streaker ED Dimitrov DS. 2012 Characterization of human IgG repertoires in an acute HIV-1 infection. Exp Mol Pathol. 93:399–407. 23036472 10.1016/j.yexmp.2012.09.022 PMC3663482

[msw015-B10] DeKosky BJ Ippolito GC Deschner RP Lavinder JJ Wine Y Rawlings BM Varadarajan N Giesecke C Dörner T Andrews SF, 2013 High-throughput sequencing of the paired human immunoglobulin heavy and light chain repertoire. Nat Biotechnol. 31:166–169. 23334449 10.1038/nbt.2492 PMC3910347

[msw015-B11] DeKosky BJ Kojima T Rodin A Charab W Ippolito GC Ellington AD Georgiou G. 2015 In-depth determination and analysis of the human paired heavy- and light-chain antibody repertoire. Nat Med. 21:86–91. 25501908 10.1038/nm.3743

[msw015-B12] Doria-Rose NA Schramm CA Gorman J Moore PL Bhiman JN DeKosky BJ Ernandes MJ Georgiev IS Kim HJ Pancera M, 2014 Developmental pathway for potent V1V2-directed HIV-neutralizing antibodies. Nature 509:55–62. 24590074 10.1038/nature13036 PMC4395007

[msw015-B13] Dunn-Walters DK Belelovsky A Edelman H Banerjee M Mehr R. 2002 The dynamics of germinal centre selection as measured by graph-theoretical analysis of mutational lineage trees. Dev Immunol. 9:233–243. 15144020 10.1080/10446670310001593541 PMC2276115

[msw015-B14] Dunn-Walters DK Spencer J. 1998 Strong intrinsic biases towards mutation and conservation of bases in human IgVH genes during somatic hypermutation prevent statistical analysis of antigen selection. Immunology 95:339. 9824495 10.1046/j.1365-2567.1998.00607.x PMC1364398

[msw015-B15] Edgar RC. 2004 MUSCLE: multiple sequence alignment with high accuracy and high throughput. Nucleic Acids Res. 32:1792–1797. 15034147 10.1093/nar/gkh340 PMC390337

[msw015-B16] Elhanati Y Sethna Z Marcou Q Callan CG Mora T Walczak AM. 2015 Inferring processes underlying B-cell repertoire diversity. Philos Trans R Soc Lond B Biol Sci. 370:20140243. 26194757 10.1098/rstb.2014.0243 PMC4528420

[msw015-B17] Felsenstein J. 1981 Evolutionary trees from DNA sequences: a maximum likelihood approach. J Mol Evol. 17:368–376. 7288891 10.1007/BF01734359

[msw015-B18] Frost SDW Murrell B Hossain ASMM Silverman GJ Pond SLK. 2015 Assigning and visualizing germline genes in antibody repertoires. Philos Trans R Soc Lond B Biol Sci. 370:20140240. 26194754 10.1098/rstb.2014.0240 PMC4528417

[msw015-B19] Furlong RF Yang Z. 2008 Diversifying and purifying selection in the peptide binding region of DRB in mammals. J Mol Evol. 66:384–394. 18347751 10.1007/s00239-008-9092-6

[msw015-B20] Gadagkar SR Rosenberg MS Kumar S. 2005 Inferring species phylogenies from multiple genes: concatenated sequence tree versus consensus gene tree. J Exp Zool B Mol Dev Evol. 304B:64–74. 15593277 10.1002/jez.b.21026

[msw015-B21] Gadala-Maria D Yaari G Uduman M Kleinstein SH. 2015 Automated analysis of high-throughput B-cell sequencing data reveals a high frequency of novel immunoglobulin V gene segment alleles. Proc Natl Acad Sci U S A. 112:E862–E870. 25675496 10.1073/pnas.1417683112 PMC4345584

[msw015-B22] Gaeta BA Malming HR Jackson KJL Bain ME Wilson P Collins AM. 2007 iHMMune-align: hidden Markov model-based alignment and identification of germline genes in rearranged immunoglobulin gene sequences. Bioinformatics 23:1580–1587. 17463026 10.1093/bioinformatics/btm147

[msw015-B23] Galson JD Clutterbuck EA Trück J Ramasamy MN Münz M Fowler A Cerundolo V Pollard AJ Lunter G Kelly DF. 2015 BCR repertoire sequencing: different patterns of B-cell activation after two meningococcal vaccines. Immunol Cell Biol. 93:885–895. 25976772 10.1038/icb.2015.57 PMC4551417

[msw015-B24] Galson JD Pollard AJ Trück J Kelly DF. 2014 Studying the antibody repertoire after vaccination: practical applications. Trends Immunol. 35:319–331. 24856924 10.1016/j.it.2014.04.005

[msw015-B25] Georgiou G Ippolito GC Beausang J Busse CE Wardemann H Quake SR. 2014 The promise and challenge of high-throughput sequencing of the antibody repertoire. Nat Biotechnol. 32:158–168. 24441474 10.1038/nbt.2782 PMC4113560

[msw015-B26] Giudicelli V Chaume D Lefranc M-P. 2005 IMGT/GENE-DB: a comprehensive database for human and mouse immunoglobulin and T cell receptor genes. Nucleic Acids Res. 33:D256–D261. 15608191 10.1093/nar/gki010 PMC539964

[msw015-B27] Greiff V Bhat P Cook SC Menzel U Kang W Reddy ST. 2015 A bioinformatic framework for immune repertoire diversity profiling enables detection of immunological status. Genome Med. 7:49. 26140055 10.1186/s13073-015-0169-8 PMC4489130

[msw015-B28] Grenfell BT Pybus OG Gog JR Wood JLN Daly JM Mumford JA Holmes EC. 2004 Unifying the epidemiological and evolutionary dynamics of pathogens. Science 303:327–332. 14726583 10.1126/science.1090727

[msw015-B29] Haynes BF Kelsoe G Harrison SC Kepler TB. 2012 B-cell-lineage immunogen design in vaccine development with HIV-1 as a case study. Nat Biotechnol. 30:423–433. 22565972 10.1038/nbt.2197 PMC3512202

[msw015-B30] Hershberg U Luning Prak ET. 2015 The analysis of clonal expansions in normal and autoimmune B cell repertoires. Philos Trans R Soc Lond B Biol Sci. 370:20140239. 26194753 10.1098/rstb.2014.0239 PMC4528416

[msw015-B31] Hershberg U Uduman M Shlomchik MJ Kleinstein SH. 2008 Improved methods for detecting selection by mutation analysis of Ig V region sequences. Int Immunol. 20:683–694. 18397909 10.1093/intimm/dxn026

[msw015-B32] Hoehn KB Gall A Bashford-Rogers R Fidler SJ Kaye S Weber JN McClure MO SPARTAC Trial Investigators Kellam P Pybus OG 2015 Dynamics of immunoglobulin sequence diversity in HIV-1 infected individuals. Philos Trans R Soc Lond B Biol Sci. 370:20140241. 26194755 10.1098/rstb.2014.0241 PMC4528418

[msw015-B33] Huelsenbeck JP Bull JJ Cunningham CW. 1996 Combining data in phylogenetic analysis. Trends Ecol Evol. 11:152–158. 21237790 10.1016/0169-5347(96)10006-9

[msw015-B34] Jackson KJL Liu Y Roskin KM Glanville J Hoh RA Seo K Marshall EL Gurley TC Moody MA Haynes BF, 2014 Human responses to influenza vaccination show seroconversion signatures and convergent antibody rearrangements. Cell Host Microbe 16:105–114. 24981332 10.1016/j.chom.2014.05.013 PMC4158033

[msw015-B35] Jiang N He J Weinstein JA Penland L Sasaki S He X-S Dekker CL Zheng N-Y Huang M Sullivan M, 2013 Lineage structure of the human antibody repertoire in response to influenza vaccination. Sci Transl Med. 5(171):171ra19. 10.1126/scitranslmed.3004794 PMC3699344 23390249

[msw015-B36] Kepler TB. 2013 Reconstructing a B-cell clonal lineage. I. Statistical inference of unobserved ancestors. F1000Res. [Internet]. Available from: http://f1000research.com/articles/2-103/v1. 10.12688/f1000research.2-103.v1 PMC3901458 24555054

[msw015-B37] Kepler TB Munshaw S Wiehe K Zhang R Yu J-S Woods CW Denny TN Tomaras GD Alam SM Moody MA, 2014 Reconstructing a B-cell clonal lineage. II. Mutation, selection, and affinity maturation. B Cell Biol. 5:170. 10.3389/fimmu.2014.00170 PMC4001017 24795717

[msw015-B38] Larimore K McCormick MW Robins HS Greenberg PD. 2012 Shaping of human germline IgH repertoires revealed by deep sequencing. J Immunol. 189:3221–3230. 22865917 10.4049/jimmunol.1201303

[msw015-B39] Laserson U Vigneault F Gadala-Maria D Yaari G Uduman M Heiden JAV Kelton W Jung ST Liu Y Laserson J, 2014 High-resolution antibody dynamics of vaccine-induced immune responses. Proc Natl Acad Sci U S A. 111:4928–4933. 24639495 10.1073/pnas.1323862111 PMC3977259

[msw015-B40] Lefranc M-P Giudicelli V Ginestoux C Jabado-Michaloud J Folch G Bellahcene F Wu Y Gemrot E Brochet X Lane J, 2009 IMGT(R), the international ImMunoGeneTics information system(R). Nucleic Acids Res. 37:D1006–D1012. 18978023 10.1093/nar/gkn838 PMC2686541

[msw015-B41] Lefranc M-P Lefranc G. 2001 The Immunoglobulin FactsBook. London (United Kingdom): Academic Press.

[msw015-B42] Lemey P Kosakovsky Pond SL Drummond AJ Pybus OG Shapiro B Barroso H Taveira N Rambaut A. 2007 Synonymous substitution rates predict HIV disease progression as a result of underlying replication dynamics. PLoS Comput Biol. 3:e29. 17305421 10.1371/journal.pcbi.0030029 PMC1797821

[msw015-B43] Lemey P Minin VN Bielejec F Pond SLK Suchard MA. 2012 A counting renaissance: combining stochastic mapping and empirical Bayes to quickly detect amino acid sites under positive selection. Bioinformatics 28:3248–3256. 23064000 10.1093/bioinformatics/bts580 PMC3579240

[msw015-B44] Li A. 2004 Utilization of Ig heavy chain variable, diversity, and joining gene segments in children with B-lineage acute lymphoblastic leukemia: implications for the mechanisms of VDJ recombination and for pathogenesis. Blood 103:4602–4609. 15010366 10.1182/blood-2003-11-3857

[msw015-B45] Liao H-X Lynch R Zhou T Gao F Alam SM Boyd SD Fire AZ Roskin KM Schramm CA Zhang Z, 2013 Co-evolution of a broadly neutralizing HIV-1 antibody and founder virus. Nature 496:469–476. 23552890 10.1038/nature12053 PMC3637846

[msw015-B46] Liu Y-J Zhang J Lane PJL Chan EY-T Maclennan ICM. 1991 Sites of specific B cell activation in primary and secondary responses to T cell-dependent and T cell-independent antigens. Eur J Immunol. 21:2951–2962. 1748148 10.1002/eji.1830211209

[msw015-B47] Lu DR Tan Y-C Kongpachith S Cai X Stein EA Lindstrom TM Sokolove J Robinson WH. 2014 Identifying functional anti-*Staphylococcus aureus* antibodies by sequencing antibody repertoires of patient plasmablasts. Clin Immunol. 152:77–89. 24589749 10.1016/j.clim.2014.02.010 PMC4066023

[msw015-B49] Martins AJ Tsang JS. 2014 Random yet deterministic: convergent immunoglobulin responses to influenza. Trends Microbiol. 22:488–489. 25179798 10.1016/j.tim.2014.07.005 PMC4961090

[msw015-B50] McCoy CO Bedford T Minin VN Bradley P Robins H Matsen FA. 2015 Quantifying evolutionary constraints on B-cell affinity maturation. Philos Trans R Soc Lond B Biol Sci. 370:20140244. 26194758 10.1098/rstb.2014.0244 PMC4528421

[msw015-B51] Mora T Walczak AM Bialek W Callan CG. 2010 Maximum entropy models for antibody diversity. Proc Natl Acad Sci U S A. 107:5405–5410. 20212159 10.1073/pnas.1001705107 PMC2851784

[msw015-B52] Muramatsu M Kinoshita K Fagarasan S Yamada S Shinkai Y Honjo T. 2000 Class switch recombination and hypermutation require activation-induced cytidine deaminase (AID), a potential RNA editing enzyme. Cell 102:553–563. 11007474 10.1016/s0092-8674(00)00078-7

[msw015-B53] Murphy K Trivers P Walport M. 2008 Janeway’s immunobiology. 7th ed. New York: Garland Science.

[msw015-B54] Nylander JAA Ronquist F Huelsenbeck JP Nieves-Aldrey J. 2004 Bayesian phylogenetic analysis of combined data. Syst Biol. 53:47–67. 14965900 10.1080/10635150490264699

[msw015-B55] Parameswaran P Liu Y Roskin KM Jackson KK Dixit VP Lee J-Y Artiles K Zompi S Vargas MJ Simen BB, 2013 Convergent antibody signatures in human dengue. Cell Host Microbe 13:691–700. 23768493 10.1016/j.chom.2013.05.008 PMC4136508

[msw015-B56] Parham P. 2009 The immune system. London: Garland Science.

[msw015-B57] Parker J Tsagkogeorga G Cotton JA Liu Y Provero P Stupka E Rossiter SJ. 2013 Genome-wide signatures of convergent evolution in echolocating mammals. Nature 502:228–231. 24005325 10.1038/nature12511 PMC3836225

[msw015-B58] Peled JU Kuang FL Iglesias-Ussel MD Roa S Kalis SL Goodman MF Scharff MD. 2008 The biochemistry of somatic hypermutation. Annu Rev Immunol. 26:481–511. 18304001 10.1146/annurev.immunol.26.021607.090236

[msw015-B59] Reddy ST Ge X Miklos AE Hughes RA Kang SH Hoi KH Chrysostomou C Hunicke-Smith SP Iverson BL Tucker PW, 2010 Monoclonal antibodies isolated without screening by analyzing the variable-gene repertoire of plasma cells. Nat Biotechnol. 28:965–969. 20802495 10.1038/nbt.1673

[msw015-B60] Retter I Althaus HH Münch R Müller W. 2005 VBASE2, an integrative V gene database. Nucleic Acids Res. 33:D671–D674. 15608286 10.1093/nar/gki088 PMC540042

[msw015-B61] Robins H. 2013 Immunosequencing: applications of immune repertoire deep sequencing. Curr Opin Immunol. 25:646–652. 24140071 10.1016/j.coi.2013.09.017

[msw015-B62] Robinson WH Lindstrom TM Cheung RK Sokolove J. 2013 Mechanistic biomarkers for clinical decision making in rheumatic diseases. Nat Rev Rheumatol. 9:267–276. 23419428 10.1038/nrrheum.2013.14 PMC3673766

[msw015-B63] Sasaki S He X-S Holmes TH Dekker CL Kemble GW Arvin AM Greenberg HB. 2008 Influence of prior influenza vaccination on antibody and B-Cell responses. PLoS One 3:e2975. 18714352 10.1371/journal.pone.0002975 PMC2500171

[msw015-B64] Scheid JF Mouquet H Ueberheide B Diskin R Klein F Oliveira TYK Pietzsch J Fenyo D Abadir A Velinzon K, 2011 Sequence and structural convergence of broad and potent HIV antibodies that mimic CD4 binding. Science 333:1633–1637. 21764753 10.1126/science.1207227 PMC3351836

[msw015-B65] Shahaf G Barak M Zuckerman NS Swerdlin N Gorfine M Mehr R. 2008 Antigen-driven selection in germinal centers as reflected by the shape characteristics of immunoglobulin gene lineage trees: a large-scale simulation study. J Theor Biol. 255:210–222. 18786548 10.1016/j.jtbi.2008.08.005

[msw015-B66] Shapiro GS Aviszus K Murphy J Wysocki LJ. 2002 Evolution of Ig DNA sequence to target specific base positions within codons for somatic hypermutation. J Immunol. 168:2302–2306. 11859119 10.4049/jimmunol.168.5.2302

[msw015-B67] Shapiro GS Ellison MC Wysocki LJ. 2003 Sequence-specific targeting of two bases on both DNA strands by the somatic hypermutation mechanism. Mol Immunol. 40:287–295. 12943801 10.1016/s0161-5890(03)00101-9

[msw015-B68] Six A Mariotti-Ferrandiz E Chaara W Magadan S Pham H-P Lefranc M-P Mora T Thomas-Vaslin V Walczak AM Boudinot P. 2013 The past, present, and future of immune repertoire biology—the rise of next-generation repertoire analysis. Front Immunol. 4:413. 24348479 10.3389/fimmu.2013.00413 PMC3841818

[msw015-B69] Smith DS Creadon G Jena PK Portanova JP Kotzin BL Wysocki LJ. 1996 Di- and trinucleotide target preferences of somatic mutagenesis in normal and autoreactive B cells. J Immunol. 156:2642–2652. 8786330

[msw015-B70] Sok D Laserson U Laserson J Liu Y Vigneault F Julien J-P Briney B Ramos A Saye KF Le K, 2013 The effects of somatic hypermutation on neutralization and binding in the PGT121 family of broadly neutralizing HIV antibodies. PLoS Pathog. 9:e1003754. 24278016 10.1371/journal.ppat.1003754 PMC3836729

[msw015-B71] Stamatakis A. 2014 RAxML version 8: a tool for phylogenetic analysis and post-analysis of large phylogenies. Bioinformatics 30:1312–1313. 24451623 10.1093/bioinformatics/btu033 PMC3998144

[msw015-B72] Stern JNH Yaari G Heiden JAV Church G Donahue WF Hintzen RQ Huttner AJ Laman JD Nagra RM Nylander A, 2014 B cells populating the multiple sclerosis brain mature in the draining cervical lymph nodes. Sci Transl Med. 6(248):248ra107. 10.1126/scitranslmed.3008879 PMC4388137 25100741

[msw015-B73] Tan Y-C Blum LK Kongpachith S Ju C-H Cai X Lindstrom TM Sokolove J Robinson WH. 2014 High-throughput sequencing of natively paired antibody chains provides evidence for original antigenic sin shaping the antibody response to influenza vaccination. Clin Immunol. 151:55–65. 24525048 10.1016/j.clim.2013.12.008 PMC4006370

[msw015-B74] Tan Y-C Kongpachith S Blum LK Ju C-H Lahey LJ Lu DR Cai X Wagner CA Lindstrom TM Sokolove J, 2014 Barcode-enabled sequencing of plasmablast antibody repertoires in rheumatoid arthritis. Arthritis Rheumatol. 66:2706–2715. 24965753 10.1002/art.38754 PMC4560105

[msw015-B75] Teng G Papavasiliou FN. 2007 Immunoglobulin somatic hypermutation. Annu Rev Genet. 41:107–120. 17576170 10.1146/annurev.genet.41.110306.130340

[msw015-B76] Thammavongsa V Kim HK Missiakas D Schneewind O. 2015 Staphylococcal manipulation of host immune responses. Nat Rev Microbiol. 13:529–543. 26272408 10.1038/nrmicro3521 PMC4625792

[msw015-B77] Trück J Ramasamy MN Galson JD Rance R Parkhill J Lunter G Pollard AJ Kelly DF. 2015 Identification of antigen-specific B Cell receptor sequences using public repertoire analysis. J Immunol. 194:252–261. 25392534 10.4049/jimmunol.1401405 PMC4272858

[msw015-B78] Uduman M Shlomchik MJ Vigneault F Church GM Kleinstein SH. 2014 Integrating B cell lineage information into statistical tests for detecting selection in Ig sequences. J Immunol. 192:867–874. 24376267 10.4049/jimmunol.1301551 PMC4363135

[msw015-B79] Victora GD Nussenzweig MC. 2012 Germinal centers. Annu Rev Immunol. 30:429–457. 22224772 10.1146/annurev-immunol-020711-075032

[msw015-B80] Volz EM Koelle K Bedford T. 2013 Viral phylodynamics. PLoS Comput Biol. 9:e1002947. 23555203 10.1371/journal.pcbi.1002947 PMC3605911

[msw015-B81] von Büdingen H-C Kuo TC Sirota M van Belle CJ Apeltsin L Glanville J Cree BA Gourraud P-A Schwartzburg A Huerta G, 2012 B cell exchange across the blood-brain barrier in multiple sclerosis. J Clin Invest. 122:4533–4543. 23160197 10.1172/JCI63842 PMC3533544

[msw015-B82] Wang C Liu Y Cavanagh MM Saux SL Qi Q Roskin KM Looney TJ Lee J-Y Dixit V Dekker CL, 2014 B-cell repertoire responses to varicella-zoster vaccination in human identical twins. Proc Natl Acad Sci U S A. 112(2):500–505. 25535378 10.1073/pnas.1415875112 PMC4299233

[msw015-B83] Wang C Liu Y Xu LT Jackson KJL Roskin KM Pham TD Laserson J Marshall EL Seo K Lee J-Y, 2014 Effects of aging, cytomegalovirus infection, and EBV infection on human B cell repertoires. J Immunol. 192:603–611. 24337376 10.4049/jimmunol.1301384 PMC3947124

[msw015-B84] Wu X Yang Z-Y Li Y Hogerkorp C-M Schief WR Seaman MS Zhou T Schmidt SD Wu L Xu L, 2010 Rational design of envelope identifies broadly neutralizing human monoclonal antibodies to HIV-1. Science 329:856–861. 20616233 10.1126/science.1187659 PMC2965066

[msw015-B85] Wu X Zhang Z Schramm CA Joyce MG Do Kwon Y Zhou T Sheng Z Zhang B O’Dell S McKee K, 2015 Maturation and diversity of the VRC01-antibody lineage over 15 years of chronic HIV-1 infection. Cell 161:470–485. 25865483 10.1016/j.cell.2015.03.004 PMC4706178

[msw015-B86] Xu JL Davis MM. 2000 Diversity in the CDR3 region of VH is sufficient for most antibody specificities. Immunity 13:37–45. 10933393 10.1016/s1074-7613(00)00006-6

[msw015-B87] Yaari G Benichou JIC Vander Heiden JA Kleinstein SH Louzoun Y. 2015 The mutation patterns in B-cell immunoglobulin receptors reflect the influence of selection acting at multiple time-scales. Philos Trans R Soc Lond B Biol Sci. 370:20140242. 26194756 10.1098/rstb.2014.0242 PMC4528419

[msw015-B88] Yaari G Kleinstein SH. 2015 Practical guidelines for B-cell receptor repertoire sequencing analysis. Genome Med. 7: Article 121. 10.1186/s13073-015-0243-2 PMC4654805 26589402

[msw015-B89] Yaari G Uduman M Kleinstein SH. 2012 Quantifying selection in high-throughput immunoglobulin sequencing data sets. Nucleic Acids Res. 40(17):e134. 22641856 10.1093/nar/gks457 PMC3458526

[msw015-B90] Yaari G Vander Heiden JA Uduman M Gadala-Maria D Gupta N Stern JNH O’Connor KC Hafler DA Laserson U Vigneault F, 2013 Models of somatic hypermutation targeting and substitution based on synonymous mutations from high-throughput immunoglobulin sequencing data. Front Immunol. 4: Article 358. 10.3389/fimmu.2013.00358 PMC3828525 24298272

[msw015-B91] Yang Z Swanson WJ. 2002 Codon-substitution models to detect adaptive evolution that account for heterogeneous selective pressures among site classes. Mol Biol Evol. 19:49–57. 11752189 10.1093/oxfordjournals.molbev.a003981

[msw015-B92] Ye J Ma N Madden TL Ostell JM. 2013 IgBLAST: an immunoglobulin variable domain sequence analysis tool. Nucleic Acids Res. 41:W34–W40. 23671333 10.1093/nar/gkt382 PMC3692102

[msw015-B93] Zhang J Kumar S. 1997 Detection of convergent and parallel evolution at the amino acid sequence level. Mol Biol Evol. 14:527–536. 9159930 10.1093/oxfordjournals.molbev.a025789

[msw015-B94] Zhou T Zhu J Wu X Moquin S Zhang B Acharya P Georgiev IS Altae-Tran HR Chuang G-Y Joyce MG, 2013 Multidonor analysis reveals structural elements, genetic determinants, and maturation pathway for HIV-1 neutralization by VRC01-Class antibodies. Immunity 39:245–258. 23911655 10.1016/j.immuni.2013.04.012 PMC3985390

[msw015-B95] Zhu J Ofek G Yang Y Zhang B Louder MK Lu G McKee K Pancera M Skinner J Zhang Z, 2013 Mining the antibodyome for HIV-1-neutralizing antibodies with next-generation sequencing and phylogenetic pairing of heavy/light chains. Proc Natl Acad Sci U S A. 110:6470–6475. 23536288 10.1073/pnas.1219320110 PMC3631616

